# 215. Trends in Syphilis and the Impact of the COVID-19 Pandemic in Florida: A multiscale joinpoint regression analysis

**DOI:** 10.1093/ofid/ofae631.073

**Published:** 2025-01-29

**Authors:** Evan Niu, Rachel Sareli, Paula A Eckardt, Candice Sareli, Jianli Niu

**Affiliations:** Pine Crest School, Fort Lauderdale, FL; Pine Crest School, Fort Lauderdale, FL; Memorial Healthcare System, Hollywood, Florida; Memorial Healthcare System, Hollywood, Florida; Memorial Healthcare System, Hollywood, Florida

## Abstract

**Background:**

Syphilis, a sexually transmitted infection caused by the bacterium *Treponema pallidum*, has been increasing in incidence both locally and globally. Visualizing and analyzing time series trends of syphilis across different demographics and geographic locations, and how they varied during the COVID-19 pandemic, would help to develop strategies to address the rising syphilis rates.

**Figure 1.**
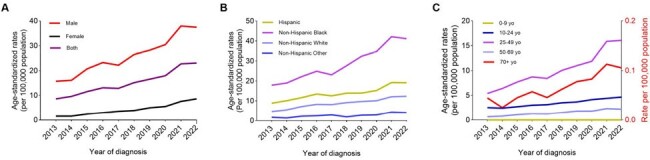
Trends of age-standardized syphilis rates in Florida, 2013 - 2022, stratified by sex, racial and age groups. (A) ASSR by sex; (B) ASSR by racial groups; (C) ASSR by age groups. Ages 10 - 24, 25 - 49, and 50 - 69 years are depicted on the left axis, while ages 0 - 9 and 70+ years are shown on the right y-axis. ASSR, age-standardized syphilis rates.

**Methods:**

Ten years of surveillance data (2013–2022) were extracted from the Florida Department of Health. Average annual percentage changes (AAPC) in age-standardized syphilis rates (ASSR) were analyzed according to age, sex, race, geographic locations, and the COVID-19 pandemic period using joinpoint regression models.

**Figure 2.**
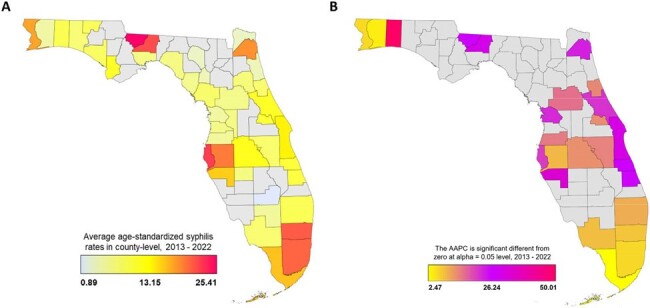
Geographic distribution of syphilis cases in Florida, 2013 - 2022. (A) The ASSR at county levels in each year was averaged from 2013 to 2022 and the averaged ASSR in each county is mapped. (B) The AAPC across the entire 10-year period in each county is mapped. ASSR, age-standardized syphilis rates; AAPC, average annual percentage change.

**Results:**

The ASSR significantly increased from 2013 to 2022 across different demographic groups, with an overall AAPC of 11.46 (95% CI 9.85–13.43) (Figure 1). The increase was greater in females, with an AAPC of 20.97 (95% CI, 18.61–24.49), compared to males with an AAPC of 10.34 (95% CI 8.19–12.98). The increasing trend was observed across all age groups, with greater increase among individuals aged 25–49 years (AAPC=12.32, 95% CI 10.09–15.18), aged 50–69 years (AAPC=13.42, 95% CI 9.41–18.89), and aged over 70 years (AAPC=13.63, 95% CI 9.23–21.95), compared to those aged ≤ 24 years (AAPC=7.86, 95% CI 7.06–8.81). The increasing trends were comparable across race groups, with an AAPC of 8.08 (95% CI 5.47–11.15) for Hispanic, 11.84 (95% CI 10.02–14.09) for non-Hispanic White, 10.49 (95% CI 8.75–12.66) for non-Hispanic Black, and 11.29 (95% CI 5.28–19.57) for non-Hispanic other races, respectively. There was an upward trend in ASSR during the COVID-19 pandemic (AAPC=12.99, 95% CI 8.48–16.21) compared to the pre-pandemic period (AAPC=11.58, 95% CI 10.17–12.76), but the difference in AAPC between the two periods did not reach statistical significance. The ASSR and the AAPC varied greatly among counties (Figure 2).

**Conclusion:**

The incidence trend of syphilis increases substantially from 2013 to 2022, with differences observed among age, gender, and geographies in Florida. The COVID-19 pandemic produced an increasing incidence of syphilis. Efforts are needed to implement strategies to address the rising syphilis rates within high-incidence groups and communities across the state.

**Disclosures:**

**All Authors**: No reported disclosures

